# Coastal connectivity and spatial subsidy from a microbial perspective

**DOI:** 10.1002/ece3.2408

**Published:** 2016-08-29

**Authors:** Christin Säwström, Glenn A. Hyndes, Bradley D. Eyre, Megan J. Huggett, Matthew W. Fraser, Paul S. Lavery, Paul G. Thomson, Flavia Tarquinio, Peter D. Steinberg, Bonnie Laverock

**Affiliations:** ^1^ School of ScienceCentre for Marine Ecosystems ResearchEdith Cowan UniversityJoondalupWAAustralia; ^2^ School of Environment Science and EngineeringCentre for Coastal Biogeochemistry ResearchSouthern Cross UniversityLismoreNSWAustralia; ^3^ The UWA Oceans Institute and the School of Plant BiologyThe University of Western AustraliaCrawleyWAAustralia; ^4^ The School of Civil, Environmental and Mining Engineering and The UWA Oceans InstituteThe University of Western AustraliaCrawleyWAAustralia; ^5^ Sydney Institute of Marine ScienceMosmanNSWAustralia; ^6^ Centre for Marine Bio‐Innovation and School of Biological, Earth and Environmental SciencesUniversity of New South WalesSydneyNSWAustralia; ^7^ Plant Functional Biology and Climate Change ClusterUniversity of Technology SydneySydneyNSWAustralia

**Keywords:** coastal connectivity, conceptual model, microbial activity, organic matter, remineralization, respiration, spatial subsidy

## Abstract

The transfer of organic material from one coastal environment to another can increase production in recipient habitats in a process known as spatial subsidy. Microorganisms drive the generation, transformation, and uptake of organic material in shallow coastal environments, but their significance in connecting coastal habitats through spatial subsidies has received limited attention. We address this by presenting a conceptual model of coastal connectivity that focuses on the flow of microbially mediated organic material in key coastal habitats. Our model suggests that it is not the difference in generation rates of organic material between coastal habitats but the amount of organic material assimilated into microbial biomass and respiration that determines the amount of material that can be exported from one coastal environment to another. Further, the flow of organic material across coastal habitats is sensitive to environmental change as this can alter microbial remineralization and respiration rates. Our model highlights microorganisms as an integral part of coastal connectivity and emphasizes the importance of including a microbial perspective in coastal connectivity studies.

## 1 Introduction

The cross‐habitat movement of organic material, nutrients, and macro‐ and microorganisms provides an important ecosystem process that shapes food webs and influences productivity in all ecosystems. A “spatial subsidy” describes the movement of material and organisms from a donor system, which subsequently increases productivity and/or shifts biodiversity in a recipient system (Polis, Anderson, & Holt, 1997). Spatial subsidies most often occur when material from a productive donor system is imported into a recipient system with limited in situ productivity (Polis et al., 1997). The concept of spatial subsidies has led to the broader recognition that coastal ecosystems are heterogeneous habitats connected through material and nutrient exchanges through the movement of dissolved or particulate organic matter, or nekton (Hyndes et al., 2014).

The connectivity between coastal habitats is mediated by microorganisms and their actions in organic matter cycling (Azam & Malfatti, 2007; Koho et al., 2013; Rivkin & Legendre, 2001). Dissolved organic matter (DOM) and particulate organic matter (POM) provide growth substrates for microorganisms, which then mediate the transfer of material and nutrients from one coastal ecosystem to another, but the importance of each organic substrate differs among ecosystems (Azam & Malfatti, 2007; Hyndes et al., 2014). For example, POM is an important source of organic material transported from kelp forests, while DOM is the main source of material exported from salt marsh ecosystems (Hyndes et al., 2014). Spatial subsidies between different coastal environments will be mediated by both the decomposition (remineralization) of POM by attached microbial communities, and the subsequent assimilation and recycling of DOM by attached and free‐living microorganisms in recipient habitats (Azam & Malfatti, 2007). However, understanding of the importance of these microbially mediated pathways for organic material transfer across different coastal ecosystems is limited.

The extent of a spatial subsidy depends on a range of factors, including: the amount of organic material being transported; the transport rate (and therefore the amount of time available for microbial transformation) of the organic material; the productivity and edge‐to‐area ratios of the recipient system; and the permeability of the ecosystem boundary (Hyndes et al., 2014; Polis et al., 1997). The capacity of recipient systems to uptake and recycle allochthonous (i.e., imported) organic material will strongly influence the impact those materials have on the productivity and biodiversity of recipient systems. The role of microorganisms in cycling organic material has been well studied in coastal habitats (Azam & Malfatti, 2007 and references within), but the form of organic material being released from different coastal habitats varies (Hyndes et al., 2014), suggesting that their role differs across habitats in coastal environments. Despite the growing evidence of connectivity across a range of coastal environments through the movement of organic material (Hyndes et al., 2014), the role of microbial processes in facilitating this coastal connectivity remains a key knowledge gap.

**Figure 1:**
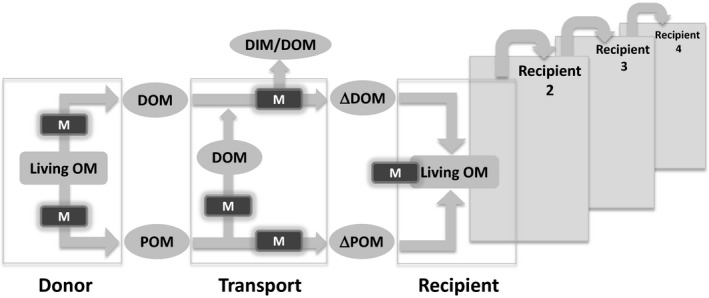
The conceptual model of microbially mediated flow of organic matter between donor and recipient coastal habitats. Living OM, living organic matter; M, site of microbial action; POM, particulate organic matter; DOM, dissolved organic matter; DIM, dissolved inorganic matter; ΔPOM, transformed POM; ΔDOM, transformed DOM

In this synthesis paper from published literature, we develop a conceptual model of the role of microorganisms in facilitating spatial subsidies across shallow coastal environments through the generation, transfer, and uptake of DOM and POM. As microbial processes can differ across habitats (e.g., Apprill & Rappé, 2011; Clasen & Shurin, 2015; Egan et al., 2012; Robertson, Mills, & Zieman, 1982; Van Oevelen, Middelburg, Soetaert, & Moodley, 2006), their role in facilitating the transfer and uptake of organic material across coastal ecosystems is likely to differ. We therefore discuss the conceptual model in relation to a range of hard and soft substrate habitats (e.g., coral reefs and kelp beds vs. sea grass meadows, salt marshes, and mangroves) to illustrate how microbial activities underpin the generation, transfer, and uptake of materials in shallow coastal environments, and also how their roles are likely to differ across habitats (Fig. 1). Given that the strength of connectivity between coastal environments is likely to be affected by changes through human disturbances, we also discuss the influence of key environmental variables on microbial processes and how changes in ocean temperatures, acidification, and nutrient loads are likely to influence the cycling of organic material and connectivity in coastal ecosystems.

## 2 A Conceptual Model for Coastal Connectivity Mediated by Microorganisms

**Figure 2:**
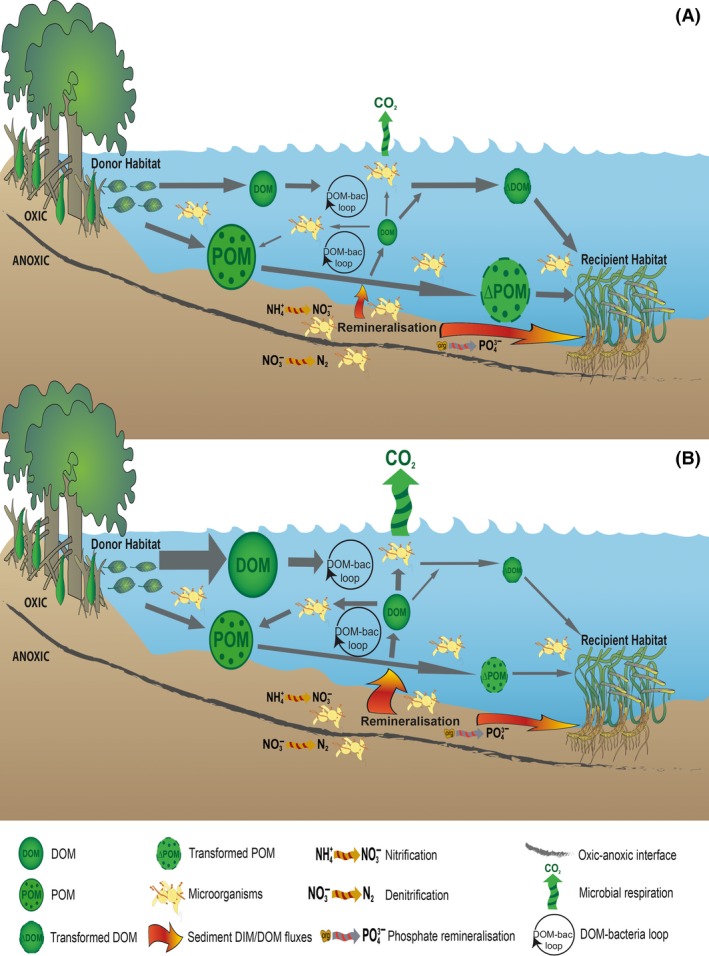
(A) Microbially mediated exchange and transfer of matter between mangrove (donor habitat) and sea grass (recipient habitat) (B) hypothesized alterations to microbially mediated exchange and transfer of matter with environmental change (altered temperature and nutrient regimes). The gray arrows indicate direction of flow from the various components of the model, and the size of the symbol reflects the relative size of the pool or process

Our conceptual model is based on microorganisms influencing connectivity in shallow coastal ecosystems during three phases: (1) generation of organic material available for transport from donor habitats; (2) transportation and associated microbial transformations of organic material; and (3) deposition and uptake of organic material within the recipient habitat (Figs 1 and 2A). This process does not necessarily end with the incorporation of material into the recipient habitat, as the recipient habitat may subsequently act as a donor habitat through the release of material, thus continuing the cycle (Fig. 1).

### 2.1 Generation of organic material in donor habitats

Coastal systems generate a broad range of organic material, but material from a donor habitat can be broadly classified into two categories: (1) POM, with a broad size range from whole kelp thalli to particles that are retained by a filter with a pore size between 0.22 and 0.7 μm; and (2) DOM that is widely defined as material passing through a given filter pore size (between 0.22 and 0.7 μm) (Volkman & Tanoue, 2002). This pool of organic material is constantly being transformed and taken up by microorganisms (Azam & Malfatti, 2007), with most being lost as CO_2_ but the remaining fraction becoming available for advection to adjacent habitats (Table 1).

**Table 1:** Mean generation rates of particulate and dissolved organic matter (POM and DOM), burial and respiration rates in hard and soft substrate coastal habitats

Habitat	POM/DOM generation rate (g C m^−2^ day^−1^)		Burial rate (g C m^−2^ day^−1^)	Respiration rate(g C m^−2^ day^−1^)
	POM	DOM		
Seaweed	0.8^a^	0.4^b^		5.8^c^
Mangroves	0.5^a^	0.3^d^	0.4^e^	5.1^c^
Salt marshes	0.2^f^	0.3^f^	0.6^e^	5.5^c^
Coral reefs	0.1^g^	0.4^g^		4.3^c^
Sea grasses	0.3^a^	0.1^b^	0.4^e^	1.9^c^
Sediments	0.2^b^	0.03^f^	0.001^h^	0.03^c^^,^ i

Most hard substrate habitats such as seaweed and coral reefs do not bury carbon as the hard substratum makes burial impossible (Duarte et al., 2013).

a Cebrian (2002).

b Barrón et al. (2012).

c Middelburg, Duarte, and Gattuso (2005).

d Maher et al. (2013).

e Duarte et al. (2013).

f Maher and Eyre (2010).

g Nakajima et al. (2010).

h Oakes and Eyre (2014). Estimated from ^13^C‐bicarbonate incorporation into sediments.

i Using the exponential relationship between sediment respiration versus depth (Middelburg et al., 2005); Respiration = 32e^−0.0077*z*^, where *z* is water depth (m), we are including shallow coastal habitats down to 20 m water depth.

Particulate organic matter can comprise a complex mixture of living and nonliving organic material, ranging in size from small colloids to large aggregates, and often forms hotspots (e.g., marine snow) for microorganisms, offering sites of elevated microbial biomass, anoxic microenvironments, and accelerated metabolism (Lehto, Glud, á Norði, Zhang, & Davison, 2014; Volkman & Tanoue, 2002). The main sources contributing to the POM pool in coastal areas are phytoplankton, macrophytes, macroalgae, and inflow of terrestrial organic matter (Volkman & Tanoue, 2002). The DOM pool can be generated from phytoplankton exudation, leachates from live and senescent macrophytes, viral lysis, sloppy feeding by metazoan grazers, and POM solubilization by prokaryotic ectoenzymes (Azam & Malfatti, 2007). The biological availability (or lability) of the DOM pool largely determines how much of it can be exported. Labile DOM is likely to be used in hours–days within the donor habitat, while more refractory DOM is more likely to be exported to, and therefore subsidize, a recipient habitat (Guillemette & del Giorgio, 2012). Furthermore, some of the DOM pool is converted into new microbial biomass via the microbial loop and therefore contributes to the POM pool (Azam et al., 1983).

### 2.2 Transportation and transformation of organic material

Once exported, the transportation of both the dissolved and particulate fractions of organic material can be a rapid or slow process depending on prevailing conditions and distance to the recipient habitat (Hyndes et al., 2014). During transportation, microbial communities assimilate and transform organic material in both the pelagic and benthic zones (Amon & Benner, 1996; Azam & Malfatti, 2007; Robertson et al., 1982; Yamada, Fukuda, Inoue, Kogure, & Nagata, 2013).

Microorganisms acting on POM enhance the leakage of DOM and can alter the nutritional quality of the particulate matter (Norderhaug, Fredriksen, & Nygaard, 2003). During transportation, material may move vertically between the pelagic and benthic habitats and be transformed several times through microbial action, with most of the organic material being respired as CO_2_ (Robertson et al., 1982; Oakes & Eyre, 2014; Azam & Malfatti, 2007; Table 1). Furthermore, refractory DOM can be generated in the microbial loop via POM degradation, direct exudation from microbial cells during production and proliferation, and viral lysis of microbial cells which release carbon and organic nutrients previously tied up as cellular materials (Jiao et al., 2010). This refractory DOM can then be available for advection to recipient habitats and may represent an important microbial generated spatial subsidy.

The capacity for microorganisms to facilitate a subsidy or donation to recipient habitats during the transportation phase will be a balance between microbial uptake rates and the rate of transportation of material to the recipient habitat. This will continue until the DOM or POM has been respired as CO_2_, incorporated into organic matter, buried in a recipient habitat, or passes through that habitat. An area of uncertainty is the relative importance of the microorganisms associated with imported material versus those in the recipient habitat for the subsequent transformation of incoming material. For particulate matter, microorganisms on the imported material may be critical to the initial release of DOM and to the nutritional quality of the material for consumers. However, the resident microbial assemblage that colonizes the incoming material may be important for the subsequent decomposition and nutritional quality of the material.

### 2.3 Uptake of organic material in recipient habitats

Once in a recipient habitat, the resident microbial community captures and transforms imported DOM. These microorganisms are important in the decomposition (remineralization) of POM and the release of inorganic material, and the subsequent alteration of the physico‐chemical environment (e.g., redox and pH) (Norderhaug et al., 2003). Burial into sediments reduces recycling of organic material, as most soft sediment habitats are low oxygen environments that are largely unfavorable for decomposition (Canfield, Kristensen, & Thamdrup, 2005). The capacity for buried organic material to be recycled depends on the ability of microorganisms to survive in deep sediments and to act on the forms of organic material that persist there (Koho et al., 2013). Microorganisms can remobilize previously buried organic material, should disturbance re‐introduce it to environments where conditions are more suitable for microbial action (e.g., resuspension of sediments during storm events). In this sense, microbial communities can facilitate a temporal trophic connectivity—one in which the productivity donated during a past time can support production in that or another habitat at a subsequent time, possibly millennia later (Caraco, Bauer, Cole, Petsch, & Raymond, 2010).

## 3 Applying the Conceptual Model to Coastal Environments

To determine the role of microorganisms in coastal connectivity processes, and how those roles may differ in different habitats, we use data from the literature on microbially facilitated organic material during the generation, transportation/transformation, and uptake of POM/DOM in a range of shallow coastal habitats. For ease of interpretation, we focus on the: (1) generation of organic material in hard versus soft substrate donor habitats, covering seaweed, coral reefs, macrophytes, and microphytobenthos (MPB); (2) transportation/transformation of organic material; and (3) uptake in the recipient habitat.

### 3.1 Generation of organic material in hard substrate habitats

Seaweeds and corals generally occur on, or form hard substrates, and are important in generating organic material. Seaweeds generate more POM than coral reefs but similar amounts of DOM (Table 1). The production of organic detrital material in temperate kelp forests averages around 2.7 g C m^−2^ day^−1^, with laminarian brown algae (kelp) generating as much as 9.6 g C m^−2^ day^−1^ (Hyndes et al., 2014). It has been estimated that 82% of global kelp production is lost as fronds or whole thalli (up to 2.2 g C m^−2^ day^−1^) but also via blade erosion and fragmentation (up to 7.4 g C m^−2^ day^−1^; de Bettignies, Wernberg, Lavery, Vanderklift, & Mohring, 2013; Krumahansl and Scheibling 1996). In temperate systems, around half of the “lost” kelp detritus can be transported and taken up as POM and/or DOM (i.e., breakdown of POM into DOM by microorganisms) in adjacent near shore habitats (Hyndes et al., 2014). There is evidence that epiphytic microorganisms can enhance the transfer of nutrients from detrital material to higher trophic levels through preferential grazing on microbially degraded kelp material (Clasen & Shurin, 2015; Norderhaug et al., 2003). In comparison, DOM and POM generation in coral reefs occurs through mucus release or mass spawning events and around 18% of the total organic carbon generated is exported from the reef to oceanic waters (Naumann et al., 2012). Spawning events can release 0.02 g C/m^2^ of egg associated carbon overnight (Apprill & Rappé, 2011), which stimulates benthic respiration and production and associated microbial driven loss and recycling of nutrients within the coral reef system (Eyre, Glud, & Pattern, 2008; Glud, Eyre, & Patten, 2008).

Both seaweeds and corals release DOM at rates of 0.4 g C m^−2^ day^−1^ (Table 1). For seaweeds, DOM is released through direct exudation and leakage during fragmentation of POM, which can account for 27% of seaweed primary production (Barrón, Apostolaki, & Duarte, 2012; Table 1), while corals primarily generate DOM via the release of mucus (comprising mostly glucose and glycoproteins; Nakajima et al., 2010; Wild, Laforsch, Mayr, Fuß, & Niggl, 2012). The release of DOM provides a constant supply of organic compounds (highly degradable carbohydrates and high protein content) in seaweed and coral habitats that can be utilized by microorganisms that can reach abundances of 10^6^ or 10^9^ cells/mm^2^ in coral tissue and seaweed, respectively (Egan et al., 2012; Rohwer, Seguritan, Azam, & Knowlton, 2002; Wild et al., 2012). Further, the microbial communities in both habitats are distinct from communities within adjacent seawater (Egan et al., 2012; Nelson, Alldredge, McCliment, Amaral‐Zettler, & Carlson, 2011). Coral mucus can be quite diverse, with up to several thousand species associated with a single coral colony, whereas microbial communities associated with seaweeds are often species‐specific (Egan et al., 2012; Rohwer et al., 2002).

### 3.2 Generation of organic material in soft substrate habitats

Most shallow coastal waters are soft substrate habitats where photoautotrophs (macrophytes and microphytobenthos [MPBs]) are able to grow on and within the sediment (Gattuso et al., 2006). Macrophytes (i.e., sea grasses, mangroves and salt marsh plants) and MPBs are generally highly productive and harbor some of the most diverse microbial communities on Earth and can produce and store large amounts of carbon (Lozupone & Knight, 2007; Macintyre, Geider, & Miller, 1996).

Soft sediment habitats often have higher rates of POM generation than DOM, and generation rates of organic carbon from macrophyte habitats to coastal oceans vary widely but average around 0.3 g C m^−2^ day^−1^. Export rates of macrophyte POM are relatively low compared to seaweeds (Cebrian, 2002; Hyndes et al., 2014; Table 1). In salt marshes, connectivity across ecosystems changes with flood and ebb tides but relies more on dissolved nutrients, with DOM generation being higher than POM (Table 1). In sea grass meadows, the primary source of dissolved organic carbon (DOC) in the water column is labile exudates from growing leaves, which account for <5% of the carbon fixed during sea grass photosynthesis (Ziegler & Benner, 1999). Sea grass meadows (*Posidonia oceanica*) in the western Mediterranean can release and export up to three times more DOM (52.3 ± 11.3 g C m^−2^ year^−1^) than POM (Barrón & Duarte, 2009). Conversely, the direct export of mangrove litter (including POM) in Queensland, Australia could be in the order of 223 g C m^−2^ year^−1^; however, only a proportion of this litter makes it all the way to adjacent coastal waters (Robertson & Daniel, 1989). In fact, only a small amount of the total mangrove production is exported as particulate organic carbon and DOC, with dissolved inorganic carbon (DIC) being the largest exported carbon source from mangroves (Maher, Sanots, Golsby‐Smith, Gleeson, & Eyre, 2013; Table 1).

The microphytobenthos exist as films or mats typically comprising microalgae (diatoms, cyanobacteria, dinoflagellates, and chlorophytes) bound with sediment in a matrix of extracellular polysaccharides (EPS) produced by the cells themselves (Macintyre et al., 1996). As these communities can reach high densities and biomass (up to 10^7^ cells/cm^3^ and 560 mg Chl *a* per m^2^, respectively) and are highly productive (up to 180 mg C m^−2^ hr^−1^), they can generate significant amounts of organic material (Macintyre et al., 1996). The MPB and EPS‐bound sediment can be suspended and exported as POM (~0.2 g C m^−2^ day^−1^) by tidal action, wind driven waves, and by bioturbation (Macintyre et al., 1996; Cebrian, 2002; Table 1). In contrast, the MPB is generally not considered a major exporter of DOM, as most dissolved carbon is recycled and retained in sediments (i.e., ~30% of ^13^C‐labeled MPB‐derived carbon still remained within sediments after 31 days) (Oakes & Eyre, 2014). MPB‐generated DOM is a labile carbon source that sediment bacteria can quickly recycle and therefore control fluxes of dissolved compounds across the sediment–water interface (Oakes & Eyre, 2014).

Macrophyte habitats can also release a substantial amount of DOM during decomposition of detrital leaf particles and whole fallen leaves (e.g., Lavery, McMahon, Weyers, Boyce, & Oldham, 2013). This is a microbially assisted process whereby microorganisms cause major changes in the composition and concentration of carbohydrates, fatty acids and lipids in leaves. This process involves three major phases: (1) soluble material is rapidly leached from the detritus with rapid growth of bacteria and increase in lipid and nitrogen levels; (2) microorganisms utilize less recalcitrant plant constituents (i.e., hemicellulose and cellulose) and bacterial numbers decrease; and (3) bacterial growth ensues and detritus decomposition occurs slowly as a result of microbial degradation of more recalcitrant material (i.e., lignin and lignocelluloses) (Kristensen, 1994). While microorganisms facilitate the production of DOM and influence its composition, they also affect the release rate from the detrital material, typically reducing the flux into adjacent water, presumably through uptake to support their own growth (Maie, Jaffé, Miyoshi, & Childers, 2006).

### 3.3 Transportation and transformations of organic material between habitats

Once exported from coastal habitats, POM and DOM can undergo transformation during the transport phase. These transformations of organic material are mediated by microorganisms in the microbial loop which form the main pathway for organic material being transformed and transported between the donor and recipient habitats. The functional capabilities of microorganisms to use organic material are dependent on their capacity to produce extracellular enzymes. Most microorganisms make use of extracellular enzymes that hydrolyze POM to a size (<600 Da) that can be transported across cell walls (Bianchi, 2011). These extracellular enzymes play an essential role in the transformation of organic material and are often referred to as “the” rate‐limiting step in remineralization (Bianchi, 2011 and references within). In addition to extracellular enzyme production, microbial communities can also change the expression of transporter genes to allow the uptake of different sources of DOM. For example, bacterioplankton in the south‐eastern USA coastal waters change their transporter gene expressions when exposed to varying sources of DOC (i.e., plant‐derived and phytoplankton‐derived DOC) (Poretsky, Sun, Mou, & Moran, 2010). This illustrates that coastal microorganisms can make use of the heterogeneous DOM pool in coastal habitats as they can alter their gene expression accordingly and use a combination of extracellular enzymes. Extracellular enzymes are also produced within microbial aggregates, for example, marine snow, which are important sites of remineralization in the water column (Canfield et al., 2005; Iversen & Ploug, 2010). Aggregates (sizes 62 to 119 μm) with attached microorganisms have different physical properties compared to the starting material of the aggregate (i.e., POM), including increased porosity and lowered settling velocities (Yamada et al., 2013). This has implications for the transport of organic material to sediments as well as the potential distance the organic material can be transported.

Detached macroalgae from rocky reefs can form large rafts of POM on the ocean's surface where it can be transported long distances (>300 km) (Hyndes et al., 2014 and references within). During the transport phase, this material undergoes decomposition and fragmentation into finer POM, and DOM is released during the microbially assisted leaching process into the water column (Kristensen, 1994). Decomposition rates are highly variable among macroalgae, with 10%–60% algal biomass lost per day (Mews, Zimmer, & Jelinski, 2006). Rates may be altered by both the chemical composition of the algae and the structure of the associated microbial communities (Wakabayashi et al., 2012). For instance, the Microbulbifer strain 6532A, isolated from the thallus of *Undaria pinnatifida*, is capable of degrading both alginate and cellulose and rapidly degrades thallus fragments into single celled detritus within a day (Wakabayashi et al., 2012). The extensive mucus layer that contains alginate on detrital *Nereocystis* and *Macrocystis* appears to encourage higher decomposition rates, while the high level of phenolic compounds and higher C:N ratio in *Fucus* appears to slow down the decomposition of this alga (Mews et al., 2006).

The DOM generated by corals is highly labile and is rapidly degraded and incorporated into new microbial biomass (~32% can be recycled on a daily basis) (Naumann et al., 2012; Rohwer et al., 2002). Recent studies have indicated that reef microbial communities can be very efficient in degrading refractory oceanic DOM relative to open ocean microbial communities (Nelson et al., 2011). This has partly been explained by the difference in microbial community composition between reef systems and the open ocean but also the possible “priming effect” (i.e., input of labile organic material can stimulate bacterial production and enhance the degradation of more refractory organic material already present) of labile coral‐derived DOM that may facilitate co‐metabolism of refractory oceanic DOM (Bianchi, 2011; Nelson et al., 2011). Thus, the efficient microbial usage of organic material within reef systems suggests that relatively little will be left for transportation to another habitat.

Compared to seaweed and coral reefs, decomposition of mangrove and salt marsh leaves is slow, partly reflecting the high lignocellulose content of these angiosperms (Kristensen, 1994 and references within). For sea grass, leachates from detrital leaves range between 27 and 92 mg DOC per g dry weight, and microbial response to these leachate products can be rapid; 80%–90% of the DOC leached from *Zostera marina* can be consumed within 24 hrs with a concomitant increase in bacterial densities and biomass (Robertson et al., 1982). However, the ability of this DOC to support microbial growth declines rapidly with the age of the leaves (Lavery et al., 2013).

Although the release of nutrients from leaves in macrophytes from soft sediments is lower than seaweeds, their release is likely to be an important component for nutrient budgets of coastal ecosystems. Leachates from marsh and sea grass leaves contain protein‐like DOM, which is labile and highly biodegradable by microorganisms (Wang, Holden, Zhang, Li, & Li, 2014). For example, DOM leached from marshes and sea grasses in Florida, USA, is considered to make significant contributions to the DOM pool to adjacent coastal waters (Stabenau, Zepp, Bartels, & Zika, 2004). Similarly, in a large sea grass dominated bay in south western Australia, DOC leaching from detached sea grass leaves was estimated to be the second largest contributor of DOC to the system (191 kg of DOC per day), between one and three orders of magnitude higher than from phytoplankton leakage and groundwater inputs (Lavery et al., 2013).

### 3.4 Uptake of organic material in recipient habitats

In the water column, organic material can be taken up, directly or indirectly via bacterially mediated processes, by thalli of seaweeds, leaves of sea grasses, and by sponges within coral reef cavities (de Goeij, Moodley, Houtekamer, Carballeira, & van Duyl, 2008; Van Engeland et al., 2011). The uptake of dissolved inorganic and organic material by seaweeds differs from that of sea grasses, which are able to exploit nutrient sources from both the sediment and the water column (Van Engeland et al., 2011). Yet, both sea grass and seaweed can directly take up small inorganic (${\text{NH}}_{4}^{+}$ and ${\text{NO}}_{3}^{-}$) and organic (urea, glycine, leucine, phenylalanine, algae‐derived DOM, and bacteria‐derived DOM) substrates with a preference for ammonium, which can be directly incorporated into amino acids (Van Engeland et al., 2011). Similarly, the encrusting sponge *Halisarca caerulea* can assimilate DOC (algal‐derived DOM and POM) in coral reefs both directly and via its associated microbial community (de Goeij et al., 2008). In intertidal sediments, carbon is taken up by the bacterial community and primarily cycled within the DOM‐bacteria loop, suggesting that bacteria themselves may act as a sink of carbon, via the cellular incorporation of organic carbon (Van Oevelen et al., 2006).

The uptake of nutrients and DOM via specific habitat‐associated microorganisms therefore facilitates production and carbon burial within the recipient system. In sea grass meadows, the uptake of dissolved inorganic and organic material may also occur via the roots and rhizomes, which are sites of high microbial activity and can be assumed the primary source of nutrients for sea grasses (Van Engeland et al., 2011).

A significant proportion of the transformed organic material within the water column is transported to coastal sediments, although loads of organic material is highly variable (Krumins, Gehlen, Arndt, Van Cappellen, & Regnier, 2013). For example, in shelf areas (<200 m), 20%–50% of the phytoplankton biomass, along with zooplankton and fecal pellets, will be deposited on the sea floor (Jørgensen, 1983), whereas macroalgae and macrophytes can be significant sources of organic material in coastal areas and contribute up to 50% of the buried carbon in marine sediments, and significantly alter microbial community structure and biogeochemical conditions in recipient habitats (Canfield et al., 2005; Duarte, Losada, Hendriks, Mazarrasa, & Marbà, 2013; Fraser, Statton, Hovey, Laverock, & Kendrick, 2016). Burial rates in coastal zones are similar to DOM/POM generation rates for these habitats (Table 1), but only a small fraction of the organic material will be buried over geological timescales, as most is lost from the system via microbial remineralization (Azam & Malfatti, 2007; Duarte et al., 2013; Table 1).

The remineralization of organic material within sediments is driven by microbial reduction–oxidation (redox) processes, where oxygen is the preferred electron acceptor during degradation at the sediment–water interface (Canfield et al., 2005). Oxygen concentrations, as well as the availability of labile organic material, are generally assumed to limit microbial processes in sediments, with oxygen being rapidly consumed in the upper few mm of coastal sediments by aerobic heterotrophs that convert high molecular weight DOC directly to CO_2_ (Bianchi, 2011; Canfield et al., 2005). Anaerobic remineralization occurs below the oxic zone and can be a considerable source of benthic DIC (as CO_2_) in coastal waters (Krumins et al., 2013). The oxic–anoxic interface is a particularly important site for the activity, diversity and abundance of microbes, and microbial community structure can change rapidly within the top few cm of the sediment (Boer et al., 2009). In undisturbed sediment, we may therefore expect to observe vertically structured microbial communities, according to the dominant metabolic process within each horizon. Microorganisms occupying the suboxic and anoxic sediment zones continue to contribute to organic matter degradation; however, rates of remineralization of organic material that becomes buried below the oxic zone are slow compared to rates at the sediment surface (Kristensen, ).

## 4 Drivers of Variation Within the Conceptual Model and Their Impact on Spatial Subsidies

From the above, we have shown that microbial processes can affect the flow of organic material within and between coastal habitats (Fig. 2A). Accordingly, changes in environmental drivers are likely to introduce significant changes to the flow of organic material, as microbial processes are sensitive to environmental change (Allison & Martiny, 2008; Vásquez‐Domínguez, Vaqué, & Gasol, 2007; Wikner & Andersson, 2012). Here, we examine the potential effects of shifts in ocean temperature, acidification and nutrients, as just three examples of environmental factors that could alter the microbially mediated flow of organic material (Fig. 2B).

### 4.1 Ocean temperature and acidification

Microbial processes are sensitive to changes in temperature, and the assimilation efficiency of organic material by microbial heterotrophs is often reduced at higher temperatures (Rivkin & Legendre, 2001). Sediment carbon mineralization rates and temperature have a strong positive correlation in freshwater systems, which suggests that warmer water leads to higher rates of organic material remineralization, and therefore lower rates of burial (Gudasz et al., 2010). In contrast, models of benthic net production in saline estuarine sediments suggest enhanced carbon burial with increasing temperatures (1–2°C increase) (Maher & Eyre, 2011). Furthermore, in coastal marine systems, increased temperatures can cause shifts in bacterial community composition and higher bacterial growth rates (Vásquez‐Domínguez et al., 2007). Increases in water temperature of 2–6°C can lead to greater consumption rates of organic material and enhancing the accumulation of DOM compared to POM (Vásquez‐Domínguez et al., 2007; Wohlers et al., 2009). This suggests that the export of POM to recipient habitats may be reduced, as bacterial organic matter remineralization and respiration in the donor system increases at higher temperatures (Vásquez‐Domínguez et al., 2007; Wohlers et al., 2009).

Increased temperatures may also enhance biofilm formation in coastal habitats, which can alter microbial community composition on the host's surface and ultimately lead to alterations in microbe–host interactions (de Oliveira et al., 2011). For example, higher temperatures led to an increase in *Bacteroides* and a reduction in *Alphaproteobacteria* in crustose coralline algae (Webster, Soo, Cobb, & Negri, 2010). Similarly, higher temperatures caused a 20%–50% increase in *Rhodobacteracae* on the brown macroalga *Fucus vesiculosus* (Stratil, Neulineger, Knecht, Friedrichs, & Wahl, 2013). Thus, temperature increases can alter both the flow of allochthonous material between ecosystems, and the uptake of DOM in recipient systems.

Compared to sea temperatures, it is less likely that ocean acidification will influence microbial remineralization rates. Most studies indicate that marine bacteria possess flexibility to cope with elevated pCO_2_ and lowered pH (Oliver, Newbold, Whiteley, & van der Gast, 2014 and references within). In fact, many microorganisms in coastal environments are tolerant of localized fluctuations in pCO_2_ and pH as these variables fluctuate widely with riverine run‐off, draw‐down by fast growing phytoplankton and microbial decomposition of organic material (Oliver et al., 2014 and references within). However, ocean acidification may result in loss of microbial habitats such as coral reefs as lowered pH causes reduced calcification rates and increased dissolution rates (Eyre, Andersson, & Cyronak, 2014).

### 4.2 Nutrient inputs

Microbial communities are crucial in nutrient cycling and show sensitivity to nutrient disturbances (i.e., N or P fertilization and C enrichment), with over 80% of nutrient addition experiments in aquatic and terrestrial environments causing an alteration in microbial community composition (Allison & Martiny, 2008). Recent studies have shown that changes in nutrient concentrations, associated with increased discharges of freshwater in coastal habitats, can shift the balance between auto‐ and heterotrophic processes (Wikner & Andersson, 2012). For example, in the northern Baltic Sea, increased nutrient concentrations in the coastal zone resulted in the carbon flow being directed toward the DOM‐bacteria loop, causing increased remineralization of DOM within the water column, thereby decreasing the POM sedimentation rates (Wikner & Andersson, 2012). Changes in nutrient concentrations also influence microbial community composition in coastal habitats. For example, in mangrove sediments, fertilization with nitrogen or phosphorus increases spatial and temporal variability in microbial communities and changes microbial production rates (Romero, Jacobson, Fuhrman, Fogel, & Capone, 2011). In tropical ecosystems such as coral reefs, bacterial community composition changes rapidly in response to terrestrial runoff and nutrient input, particularly changes in DOC and chlorophyll *a* concentrations (Witt, Wild, & Uthicke, 2012). In coral reefs, eutrophication as a result of terrestrial runoff can often cause a shift in the dominant habitat type, from corals to macroalgae which can lead to decreased bacterial diversity and the growth of “super‐heterotrophic communities” (Nelson et al., 2013). Thus, increased nutrient input through human activities is likely to significantly alter microbial communities and their ability to recycle different forms of POM and DOM in shallow coastal systems.

### 4.3 Impact of environmental drivers on microbially mediated connectivity

Based on the above, shifts in environmental drivers, such as temperature, will affect microbial metabolic activities and community composition. This may result in higher DIM/DOM than POM generation rates, but also higher transformation rates of organic material, with a larger dependence on the DOM‐bacteria loop (Fig. 2B). Further, this may increase ecosystem respiration (as microbial respiration increases) and potentially reduce the residence time of organic material within the pelagic and benthic zones due to the associated higher uptake rates (Fig. 2B). We hypothesize that ocean acidification will have a minor direct impact on microbial processes, but may result in significant indirect impacts through loss of habitats. The response of microbial communities to nutrient inputs is likely to be similar to their responses to alterations in temperature. However, one possible distinction between these drivers is that nutrient inputs have the potential to act as a “priming effect” in the system. Accordingly, the flow and transformation of organic material in the model may be enhanced, resulting in reduced transport of organic material from donor to recipient habitat as well as unlocking of refractory organic material due to the “priming effect.” Consequently, temperature and nutrient changes have the potential to cause variation to all three phases in our model, primarily by enhancing microbial uptake and transformation rates of organic material in donor and recipient habitats (Fig. 2B). One possible consequence of enhanced microbial “interception” of organic material during the transportation phase is the reduced flow of material between donor and recipient habitats, due to enhanced microbial respiration (Fig. 2B). In combination with reduced outputs of organic material due to human disturbance and loss of donor systems (Hyndes et al., 2014), alterations in microbial organic matter cycling will provide an additional and cumulative impact on spatial subsidy processes. Ultimately, this may result in reduced connectivity between coastal habitats, which would affect the supply of organic material to higher trophic levels and overall coastal productivity. Further research comparing microbial activity and the efficiency of trophic connectivity in tropical and temperate systems will improve our understanding of connectivity processes across coastal environments.

## 5 Conclusions

Our conceptualization of microbially mediated flow of organic material in shallow coastal ecosystems, based on the available literature, highlights microorganisms as important players in coastal connectivity and spatial subsidies, whose activities affect coastal habitats at a range of spatial (local to global) and temporal (minutes to millennia) scales. Our model suggests that it is not the difference in generation rates of organic material between coastal habitats, but the amount of organic material assimilated into microbial biomass and respiration that could determine the amount of material that can be exported from one coastal environment to another. Furthermore, their role in generating spatial subsidies and connecting habitats within coastal areas may be both directly and indirectly affected by environmental change, particularly by increases in temperature and nutrients. To further improve our understanding of connectivity in coastal ecosystems, we need to include data on microbial activities, diversity, community structure, and functional capabilities in investigations of coastal connectivity, as microorganisms are the first responders to environmental change and their activities can both mitigate and/or enhance the effects of these changes in our coastal ecosystems.

## Conflict of Interest

None declared.

## Funding Information

Australian Commonwealth Government's Department of Innovation, Industry, Science & Research Collaborative Research Network Program (project CRN2011:05).
